# Comment on “Enumeration of *Escherichia coli* in Probiotic Products. *Microorganisms* 2019, *7*, 437”

**DOI:** 10.3390/microorganisms8020245

**Published:** 2020-02-12

**Authors:** Trudy M. Wassenaar, Rudolf von Bünau, Kurt Zimmermann

**Affiliations:** 1 Molecular Microbiology and Genomics Consultants, Tannenstrasse 7, 55567 Zotzenheim, Germany; 2 Ardeypharm GmbH, Loerfeldstraße 20, 58313 Herdecke, Germany; buenau@pharma-zentrale.de; 3 SymbioPharm GmbH, auf dem Lüppen 10, 35745 Herborn, Germany; kurt.zimmermann@symbio.de

Recently, Zimmer and Dorea published a communication on the enumeration of *Escherichia coli* in probiotic products containing this species [1]. They investigated the content of two commercial products, Mutaflor (produced by Pharma-Zentrale GmbH, Herdecke, Germany) and Symbioflor 2 (produced by SymbioPharm GmbH, Herborn, Germany). Mutaflor is available as viable, lyophilized *E. coli* bacteria inside an acid-resistant capsule, while Symbioflor 2 contains viable *E. coli* bacteria in a liquid suspension. The authors tested the viability of the bacteria for three batches of each product. They report that the products contained *E. coli* in numbers several orders of magnitude less than claimed on the product information [1].

The authors assessed the number of viable bacteria by applying the methodology optimized for enumeration of coliform bacteria in surface waters. Serial dilutions were made in Ringers solution (the content of the Mutaflor capsules was resuspended for this) and these were enumerated in triplicate using a Colilert Quanti-train/2000 system, from which the most probable number (MPN) was obtained. This method is validated and recommended in multiple countries, including Germany [2], to estimate the number of coliform bacteria in samples expected to contain low numbers, for instance, surface water samples. For such samples the MPN methodology has been developed and optimized [3].

European producers of probiotic products are legally bound to quantify the content of their products by standardized methods, published in the European Pharmacopeia (Ph. Eur.) [4]. In the current tenth edition of the Ph. Eur., chapter 5.1.6 describes the MPN method as “developed as a means of estimating the number of viable micro-organisms present in a sample not amenable to direct plating”. That clearly does not apply to these probiotic products. Apart from direct plating or membrane filtration, for the enumeration of micro-organisms, flow cytometry or the direct epifluorescent filter technique (DEFT) are described in detail. Any alternative method must be strictly validated.

Both products are regularly tested by qualitative and quantitative methods specified in their registration documentation, in order to guarantee that they contain the number of bacteria as stated on the product. SymbioPharm performs DEFT (following DAPI staining) and fluorescence in situ hybridization (FISH) to determine the total number of bacteria (viable, viable but not culturable, and non-viable combined). The product label statement of a content of 1.5–4.5 × 10^7^ bacteria/mL is based on these methods. When serial dilutions are plated to determine the colony forming unit (CFU) content of individual Symbioflor 2 batches, this typically results in 0.3 x 10^7^ to 22.5 × 10^7^ CFU/mL, depending on the batch, tested within their shelf life. It should be noted that Symbioflor 2 is not marketed in Canada.

Mutaflor is registered in Canada as a natural health product. According to its Canadian registration, a capsule should contain 25 × 10^9^ CFU and the package obtained by the authors stated a content of “≥ 25 × 10^9^ CFU per capsule” (this is a legally permissive way to describe its content as per its registration). In contrast, the product is registered in Germany for medical use, with a requirement to contain “between 2.5 and 25 × 10^9^ CFU” per capsule. The statement on the product available in Canada was considered inaccurate by the German producer and has lately been changed to state a content of “25 × 10^9^ CFU per capsule”. When tested by the validated method as per its registration, in this case serial dilution and plating, a Mutaflor capsule typically contains between 2.5 × 10^9^ and 25 × 10^9^ CFU. 

In conclusion, the producers ensure that the stated content of both products is correct, when quantified with validated and legally binding methodology. A point to note is that even the validated quantification method can result in variable outcome. When the specified and validated agar plates for enumeration by serial dilution and plating are purchased from a different supplier, this can result in a variation in outcome of up to a factor of 100, as is reflected by other publications [5,6]. Moreover, the US pharmacopoeia USP42/NF37 gives corresponding advice in section 1223, Validation of alternative microbiological methods (Introduction): “It is extremely important in the application of this chapter that users take into account that microbiology is a logarithmic science. While we can distinguish between 100 and 1000 cfu (a difference of 1 log10), it may be not possible to discern smaller differences (less than 0.3–0.5 log10). The inherent variability of these methods is substantially greater than analytical chemistry methods.”

This demonstrates how sensitive quantitative determination of bacterial suspensions can be to experimental variation, and producers of probiotic products have to handle such variation. For this reason, they aim for the content of their product to meet the higher end of the stated quantities. After all, it is not in their interest to state higher numbers on the package than are actually present in the product.
